# Recipient of the 2006 Karen Wetterhahn Memorial Award

**Published:** 2007-01

The Superfund Basic Research Program (SBRP) is pleased to announce that Ms. Alicia R. Timme-Laragy of Duke University is the recipient of the ninth annual Karen Wetterhahn Memorial Award. The award was presented to Ms. Timme-Laragy on 12 December 2006 at the SBRP Annual Meeting in San Diego, California.

The SBRP presents this annual award to an outstanding scholar to pay tribute to the life and scientific accomplishments of Dr. Karen E. Wetterhahn, former director of the SBRP at Dartmouth College. Dr. Wetterhahn, Professor of Chemistry and the Albert Bradley Third Century Professor in the Sciences at Dartmouth College, died 8 June 1997 at age 48. Her death was the result of dimethylmercury poisoning caused by the accidental spill of a few drops of the chemical on her latex glove–covered hand. Dr. Wetterhahn was an established authority on the effects of heavy metals on biological systems as well as a dedicated teacher and mentor. She played an integral role in the administration of the sciences at Dartmouth and co-founded Dartmouth’s Women in Science Project (WISP), which is aimed at increasing the number of women majoring and taking courses in the sciences, including mathematics and engineering.

Ms. Timme-Laragy is a cum laude graduate of the Franklin & Marshall College, where she earned a B.A. in Biology and Anthropology and minored in Environmental Studies. She is in the fifth year of a Ph.D. program at Duke University, Nicholas School of the Environment and Earth Sciences, Integrated Toxicology Program, under the guidance of Dr. Richard T. Di Giulio. Ms. Timme-Laragy’s research uses a zebrafish embryo model to understand how certain combinations of PAH (polycyclic aromatic hydrocarbon) greatly increase toxicity over what is predicted from studies of single PAH. Her research is elucidating mechanisms underlying the very marked, and totally surprising, synergy that has been previously observed between PAHs that act as AHR (aryl hydrocarbon receptor) agonists and PAHs that act as CYP1A (cytochrome P450 1A) inhibitors on cardiovascular development in zebrafish embryos. According to Di Giulio, “this synergy has very important implications for risk assessments of PAHs, as currently risk estimates of this chemical class employ an additive model for summing hazards posed by individual compounds.” Understanding the mechanistic basis for this synergy will aid in a more accurate assessment of toxicity that can then be applied to other toxicants with similar mechanisms of action, and will contribute to predictive models for PAH mixtures, the environmental norm.

The NIEHS congratulates Ms. Timme-Laragy on her research accomplishments and wishes her continued success in her scientific career.

## Figures and Tables

**Figure:**
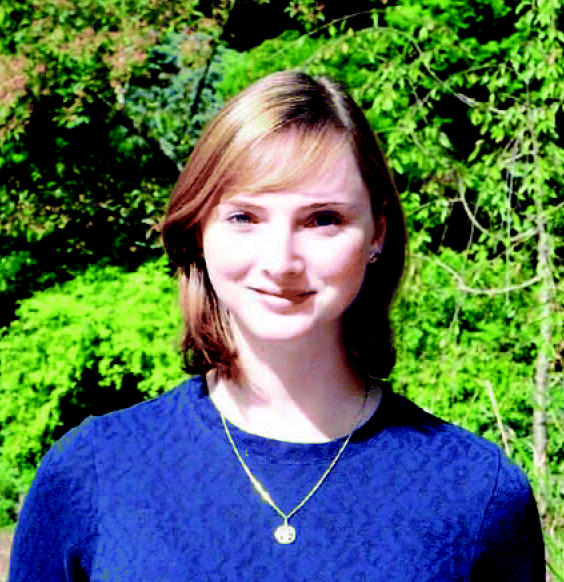
Alicia R. Timme-Laragy, Duke University

